# Telemedicine Applications for Cancer Rehabilitation: Scoping Review

**DOI:** 10.2196/56969

**Published:** 2024-08-21

**Authors:** Patricia Goncalves Leite Rocco, C Mahony Reategui-Rivera, Joseph Finkelstein

**Affiliations:** 1 Department of Biomedical Informatics School of Medicine University of Utah Salt Lake City, UT United States

**Keywords:** telerehabilitation, telemedicine, rehabilitation, cancer, exercise, physical therapy, telehealth, remote care, digital medicine, oncology, oncologist, metastases, exercising, scoping review, scoping reviews, PubMed

## Abstract

**Background:**

Cancer is a significant public health issue worldwide. Treatments such as surgery, chemotherapy, and radiation therapy often cause psychological and physiological side effects, affecting patients’ ability to function and their quality of life (QoL). Physical activity is crucial to cancer rehabilitation, improving physical function and QoL and reducing cancer-related fatigue. However, many patients face barriers to accessing cancer rehabilitation due to socioeconomic factors, transportation issues, and time constraints. Telerehabilitation can potentially overcome these barriers by delivering rehabilitation remotely.

**Objective:**

The aim of the study is to identify how telemedicine is used for the rehabilitation of patients with cancer.

**Methods:**

This scoping review followed recognized frameworks. We conducted an electronic literature search on PubMed for studies published between January 2015 and May 2023. Inclusion criteria were studies reporting physical therapy telerehabilitation interventions for patients with cancer, including randomized and nonrandomized controlled trials, feasibility studies, and usability studies. In total, 21 studies met the criteria and were included in the final review.

**Results:**

Our search yielded 37 papers, with 21 included in the final review. Randomized controlled trials comprised 47% (n=10) of the studies, with feasibility studies at 33% (n=7) and usability studies at 19% (n=4). Sample sizes were typically 50 or fewer participants in 57% (n=12) of the reports. Participants were generally aged 65 years or younger (n=17, 81%), with a balanced gender distribution. Organ-specific cancers were the focus of 66% (n=14) of the papers, while 28% (n=6) included patients who were in the posttreatment period. Web-based systems were the most used technology (n=13, 61%), followed by phone call or SMS text messaging–based systems (n=9, 42%) and mobile apps (n=5, 23%). Exercise programs were mainly home based (n=19, 90%) and included aerobic (n=19, 90%), resistance (n=13, 61%), and flexibility training (n=7, 33%). Outcomes included improvements in functional capacity, cognitive functioning, and QoL (n=10, 47%); reductions in pain and hospital length of stay; and enhancements in fatigue, physical and emotional well-being, and anxiety. Positive effects on feasibility (n=3, 14%), acceptability (n=8, 38%), and cost-effectiveness (n=2, 9%) were also noted. Functional outcomes were frequently assessed (n=19, 71%) with tools like the 6-minute walk test and grip strength tests.

**Conclusions:**

Telerehabilitation for patients with cancer is beneficial and feasible, with diverse approaches in study design, technologies, exercises, and outcomes. Future research should focus on developing standardized methodologies, incorporating objective measures, and exploring emerging technologies like virtual reality, wearable or noncontact sensors, and artificial intelligence to optimize telerehabilitation interventions. Addressing these areas can enhance clinical practice and improve outcomes for remote rehabilitation with patients.

## Introduction

Cancer is a worldwide public health problem and is the second leading cause of death in the United States [1]. Treatments for cancer, such as surgery, chemotherapy, radiation therapy, and hormone therapy, often result in psychological and physiological sequelae and side effects that interfere with treatment completion, the ability to function and perform essential daily activities, and quality of life (QoL) [2]. Physical activity is an essential component of cancer rehabilitation and effectively reduces the burden of several specific cancers, including benefits related to physical function, QoL, and cancer-related fatigue [3].

The American College of Sports Medicine concluded that exercise training is safe during and after cancer treatments and improves the QoL in several survivor groups of cancer [3]. Based on these findings, individualized and personalized programs are needed for patients with cancer depending on the type of cancer, stage of the disease, and patient goals to avoid inactivity, disability, and worsening of their QoL. Rehabilitation is a standard part of cancer care and can have the potential to reduce the burden on the health care system [4].

Unfortunately, many patients do not have access to all the cancer rehabilitation therapy due to problems related to social economics; transportation; and several other factors that impact the treatment, like work, costs, and time [5,6]. All these factors can seriously impact the patient’s access to cancer rehabilitation services in medical facilities. Conversely, technology has been growing, and treatment nowadays can be delivered to patients without the need for a face-to-face consultation [7]. This convergence of circumstances has led to the emergence of telerehabilitation, a subfield of telemedicine that uses information and communication technologies (ICTs) to develop systems capable of managing and delivering rehabilitation remotely and has been suggested as one mechanism that can reduce some barriers to accessing and providing rehabilitation [8].

Telerehabilitation has been implemented across various diseases with promising results [9-15] and was considered highly cost-effective [16,17]. Nonetheless, there is a noticeable shortage of studies evaluating the use of physical therapy in telerehabilitation for patients with cancer broadly. A review of reviews on telemedicine and digital health in patients with cancer did not uncover any documents related explicitly to rehabilitation [18]. Furthermore, the available literature reviews tend to focus on specific types of cancer [19-21], lack a systematic approach to guide the review process [22-24], target pediatric populations [25], or focus exclusively on cognitive or behavioral rehabilitation [26].

For these reasons, this scoping review aimed to identify studies regarding physical therapy telerehabilitation for survivors of cancer and understand the technology used, exercises, and outcomes of this type of treatment that has the potential to grow.

## Methods

### Study Design

This scoping review was conducted using the methodological framework of Arksey and O’Malley [27], with five major steps: (1) identify research question, (2) identify relevant studies, (3) evaluate and select studies to be included, (4) chart the data, and (5) collect, summarize, and report the results. We report this study following the PRISMA-ScR (Preferred Reporting Items for Systematic Reviews and Meta-Analyses Extension for Scoping Reviews) 2020 guidelines (Multimedia Appendix 1) [28]. The protocol was registered on the Open Science Framework [29].

### Research Question

Based on our aim, we formulated the following research question: “How are telemedicine approaches used for cancer rehabilitation?”

### Search Strategy

An electronic literature search was conducted using the PubMed database to identify relevant studies for inclusion in this scoping review. The following Boolean search terms were used: (telerehabilitation) AND (cancer) AND (“physical therapy” OR “exercise” OR “cancer rehabilitation”). No language restrictions were applied. The studies included were published between January 2015 and May 2023. This time frame was selected because, starting in 2015, global regulatory frameworks were established that promoted the use of telemedicine technologies. These frameworks provided standards and best practices, coinciding with the increased adoption of ICTs in the health care sector, thereby fostering research in this area. The literature search was reviewed and validated by an expert in telemedicine.

### Study Selection

We included studies that reported physical therapy exercises and telerehabilitation interventions for patients with cancer. Eligible designs included randomized controlled trials (RCTs) and nonrandomized controlled trials, controlled and noncontrolled before-after studies, and feasibility and usability studies that reported the intervention treatment. Exclusion criteria comprise systematic review studies and meta-analysis, no physical therapy treatment mentioned, and studies with only psychological treatment. Two reviewers (PGLR and CMR-R) conducted the selection process independently and in duplicate. Any disagreements were solved through discussion, and if consensus could not be reached, a third reviewer (JF) made the final decision.

### Data Extraction

One reviewer (CMR-R) collected the data from the documents using a predefined collection form in a Microsoft Excel spreadsheet. The other reviewer (PGLR) then double-checked the resulting form to ensure comprehensive data extraction. The data included in the study comprised the following: first author and year for each publication, type of study, specific design, sample size, sociodemographic characteristics (sex, age, race, and ethnicity), stage of cancer, and other special characteristics. Additionally, the specific technology used to deliver exercise programs or monitor each study, the type of exercise program, the description, duration, frequency, time per session, intensity of the program, and the monitoring of performance and the outcomes were charted. We synthesized findings by reporting frequencies and percentages for the abovementioned main characteristics. Furthermore, we chart the studies’ geographic location, publication date, and type of study performed in a bubble plot.

## Results

### Selection Process

**Figure 1:**
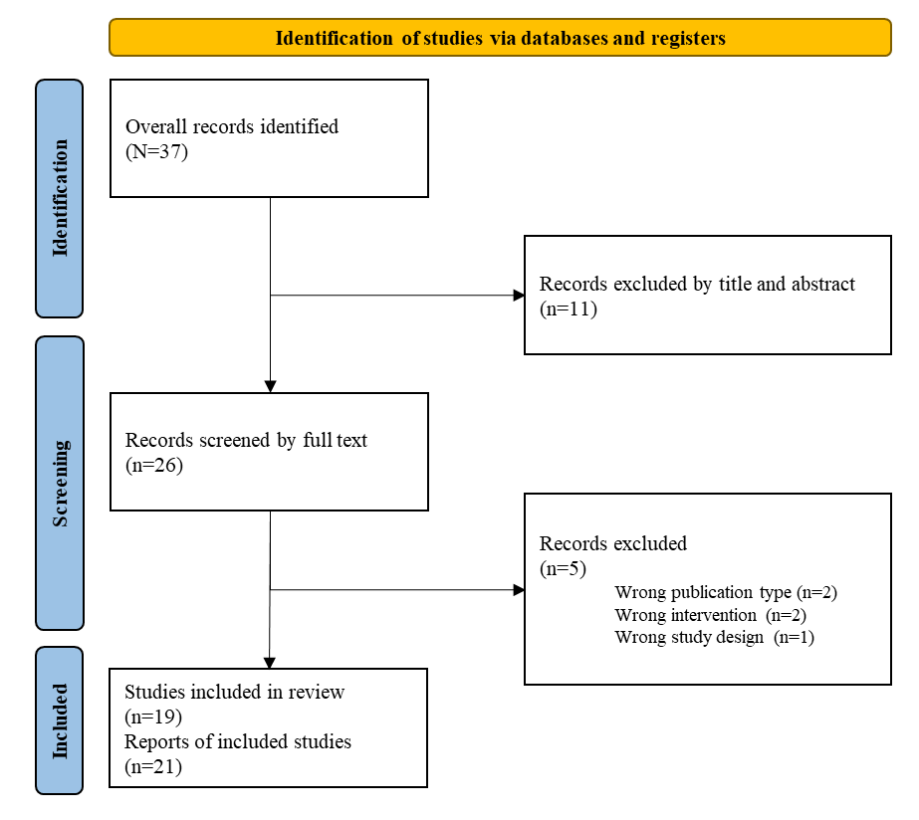
PRISMA (Preferred Reporting Items for Systematic Reviews and Meta-Analyses) flowchart of the study search and exclusion process.

Our research query provided 37 potential papers to be included in the study. After reviewing the title and abstract, we found 26 relevant documents to the research question. All these studies were then read in detail and reviewed, resulting in 21 papers to be included in the final study. This process is detailed in Figure 1.

### General Characteristics

**Figure 2:**
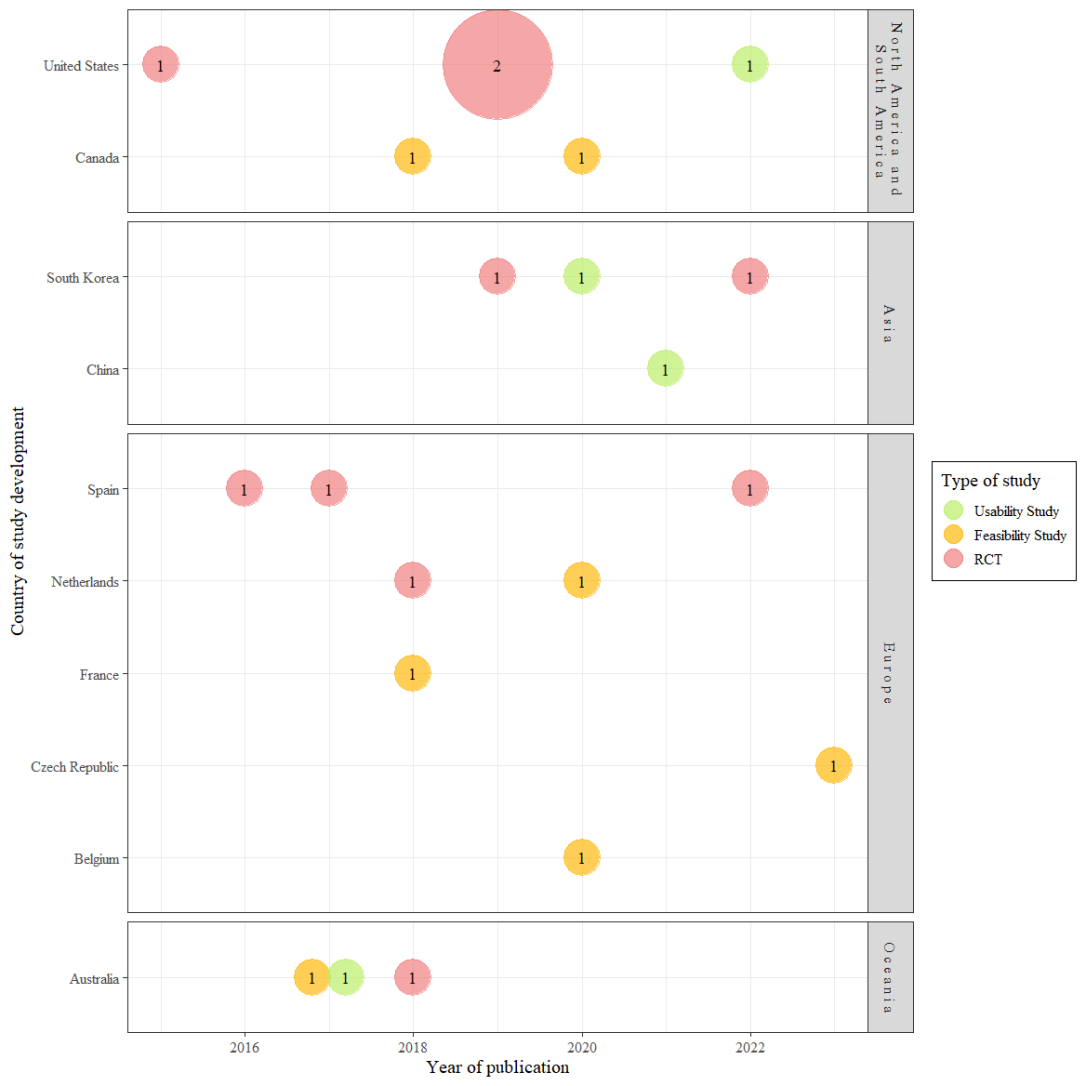
Studies by geographic location, type of study, and year of publication. RCT: randomized controlled trial.

Overall, 21 studies were included in this scoping review, spanning from 2015 to 2023 and representing a diverse range of countries and study designs. As illustrated in Figure 2, most of the papers were conducted in the United States (n=5, 24%), Spain (n=4, 19%), and South Korea (n=3, 14%). The distribution of study types across these regions shows a higher concentration of RCTs in the United States and Spain. In contrast, feasibility and usability studies were more evenly distributed across various countries.

Table 1 shows that the most common type of study was the RCT, accounting for 48% (n=10) of the included studies. Feasibility studies constituted 33% (n=7) of the studies, while usability studies comprised the remaining 19% (n=4). The specific designs of these papers varied, with many adopting a prospective approach, and evaluations were often conducted at multiple time points, typically before and after intervention. Regarding sample sizes, the total sample size for most studies was 50 or fewer, representing 57% (n=12) of the studies. Studies with sample sizes ranging from 51 to 100 comprised 33% (n=7), and only 10% (n=2) had more than 100 participants. When examining the sample size per group, 48% (n=10) of the studies had 30 or fewer participants per group, 43% (n=9) had between 31 and 50 participants per group, and only 10% (n=2) had more than 50 participants per group.

**Table 1:** Study design and participants characteristics.

Paper	Type of study	Specific design	Sample size	Participants sociodemographic characteristics	State of cancer, other special characteristics
Schwartz et al (2015) [30]	RCT^a^	Prospective, randomized, 2 arms, in a parallel group, 2-time point evaluation (pre-post)	Total=50, T^b^=25, C^c^=25	Sex: 76% (38/50) female Age: mean 52.4 (SD 12.9) years	Cancer under or after chemotherapy or radiotherapy
Galiano-Castillo et al (2016) [31]	RCT	Prospective, randomized, 2 arms, in a parallel group, 3-time point evaluation (pre-post)	Total=81, T=40, C=41	Sex: 100% female Age: T: mean 47.4 (SD 9.6) years, C: mean 49.2 (SD 7.9) years	Stage I-IIIA breast cancer after adjuvant therapy without conditions that limit exercise
Collins et al (2017) [32]	Feasibility study	Prospective, nonrandomized, 2 arms, in a parallel group, multiple time point evaluation (each appointment)	Total=30, T=15, C=15	Sex: 33.3% (10/30) female Age: T: mean 57 (range 47-77) years, C: mean 65 (range 37-72) years	Head and neck cancer under curative-intent chemotherapy or radiotherapy
Galiano-Castillo et al (2017) [33]	RCT	Prospective, randomized, 2 arms, in a parallel group, 2-time point evaluation (pre-post)	Total=81, T=40, C=41	Sex: 100% female Age: T: mean 47.4 (SD 9.6) years, C: mean 49.2 (SD 7.9) years	Stage I-IIIA breast cancer after adjuvant therapy and without conditions that limit physical exercise
Wall et al (2017) [34]	Usability study	Prospective, single-arm, 2-time point evaluation (pre-post)	Total=15	Sex: 100% male Age: mean 58.7 (range 46-70) years	Oropharyngeal squamous cell carcinoma planned for curative-intent chemotherapy without physical impairments that limit exercise
Frensham et al (2018) [35]	RCT	Prospective, randomized, 2 arms, in a parallel group, 2-time point evaluation (pre-post)	Total=91, T=46, C=45	Sex: 51.6% (47/91) female Age: T: mean 65.2 (SD 9.3) years, C: mean 66.1 (SD 9.4) years Race: White=87, Asian=2, ATSI^d^=2	Survivors of cancer who were not receiving treatment without contraindications for exercise
Gehring et al (2018) [36]	RCT	Prospective, randomized, 2 arms, in a parallel group, 2-time point evaluation (pre-post)	Total=34, T=23, C=11	Sex: 55.9% (19/34) female Age: T: mean 48.0 (SD 9.4) years, C: mean 48.0 (SD 11.9) years	Stage II-III glioma without contraindications for exercise
Vallerand et al (2018) [37]	Feasibility study	Prospective, randomized, 2 arms, in a parallel group, 2-time point evaluation (pre-post)	Total=51, T=26, C=25	Sex: 60.8% (31/51) female Age: mean 52.6 (SD 13.7) years	Leukemia, non-Hodgkin or Hodgkin lymphoma with the ability to perform exercise
Villaron et al (2018) [38]	Feasibility study	Prospective, randomized, 2 arms, in a parallel group, 2-time point evaluation (pre-post)	Total=43, T=21, C=22	Sex: 72.1% (31/43) female Age: mean 49.7 (SD 13.7) years	Cancer under chemotherapy or systemic treatment with the ability to perform exercise
Cheville et al (2019) [39]	RCT	Prospective, randomized, 3 arms, in a parallel group, 2-time point evaluation (pre-post)	Total=516, T1=72, T2=72, C=72	Sex: 49.8% (257/516) female Age: mean 65.6 (SD 11.1) years Race: White=492, non-White=24 Ethnicity: Hispanic or Latino=28	Stage IIIC or IV solid or hematologic cancer and low to moderate functional impairment that limits ambulation
Ji et al (2019) [40]	RCT	Prospective, randomized, 2 arms, in a parallel group, 2-time point evaluation (pre-post)	Total=64, T=32, C=32	Sex: 29.7% (19/64) female Age: T: mean 60.5 (SD 10.1) years, C: mean 57.9 (SD 9.8) years	Nonsmall cell lung cancer, ability to walk more than 150 m in a 6-minute walk test
Longacre et al (2019) [41]	RCT	Prospective, randomized, 3 arms, in a parallel group, 2-time point evaluation (pre-post)	Total=516, T1=172, T2=172, C=172	Sex: 49.8% (257/516) female Age: mean 65.6 (SD 11.1) years Race: White=492, non-White=24 Ethnicity: Hispanic or Latino=28	Stage IIIC or IV solid or hematologic cancer and low to moderate functional impairment that limits ambulation
van Egmond et al (2020) [42]	Feasibility study	Ambispective, 2 arms, 2-time point evaluation (pre-post)	Total=45, T=15, C=30	Sex: 26.7% (12/45) female Age: T: mean 62.8 (SD 6.9) years, C: mean 60.3 (SD 7.0) years	Esophageal or gastric cancer after surgery and with postoperative complications, with impairments that limit mobility, were assigned to in-person therapy
Kim et al (2020) [43]	Usability study	Prospective, single-arm, 3-time point evaluation (pre-during-post)	Total=31	Sex: 16.1% (5/31) female Age: mean 56.7 (SD 7.7) years	Stage I-II hepatocellular carcinoma, who could walk independently for more than 30 minutes
MacDonald et al (2020) [44]	Feasibility study	Prospective, single-arm, 2-time point evaluation (pre-post)	Total=35	Sex: 62.9% (22/35) female Age: mean 55 (SD 15.9) years	Survivors of cancer with a moderate-high disability received clearance from a physiatrist to participate in exercise
Piraux et al (2020) [45]	Feasibility study	Prospective, single-arm, 2-time point evaluation (pre-post)	Total=23	Sex: 30.4% (7/23) female Age: mean 61.7 (SD 10.6) years	Esophageal or gastric cancer planned for surgery without conditions that contraindicate or limit exercise
Zhou et al (2021) [46]	Usability study	Cross-sectional, single-arm, 1-time point evaluation (post)	Total=15	Sex: 100% female Age: mean 54.7 (SD 7.78) years	Stage I-III breast cancer after surgery, able to perform whole-body physical activity
Finkelstein et al (2022) [47]	Usability study	Cross-sectional, single-arm, 1-time point evaluation (post)	Total=11	Sex: 100% male Age: mean 68.1 (SD 11.2) years	Metastatic urogenital cancer receiving outpatient care
Lozano-Lozano et al (2020) [48]	RCT	Prospective, randomized, 2 arms, in a parallel group, 2-time point evaluation (pre-post)	Total=80, T=40, C=40	Sex: 100% female Age: T: mean 49.7 (SD 8.42) years, C: mean 53.4 (SD 8.66) years	Stage I-IIIA breast cancer, some range of ROM^e^ limitation, and overweight
Park et al (2023) [49]	RCT	Prospective, randomized, 2 arms, in a parallel group, 2-time point evaluation (pre-post)	Total=100, T=50, C=50	Sex: 100% female Age: T: mean 42.5 (SD 9.06) years, C: mean 47.3 (SD 8.55) years	Breast cancer after surgery, with limited ROM in the affected shoulder but able to perform exercise
Filakova et al (2023) [50]	Feasibility study	Prospective, single-arm, 2-time point evaluation (pre-post)	Total=11	Sex: 72.3% (8/11) female Age: mean 60.3 (SD 10) years	Lymphoma after chemotherapy with the ability to perform exercise

^a^RCT: randomized controlled trial.

^b^T=treatment group.

^c^C=control group.

^d^ATSI: Aboriginal or Torres Strait Islander.

^e^ROM: range of motion.

### Participants Characteristics

Table 1 reveals that the gender distribution among the studies was varied. Only 2 (10%) studies included all men, whereas 7 (33%) studies had more men than women. Similarly, 7 (33%) studies had more women than men, and 5 (24%) studies included all women participants. Most of the studies involved participants aged 65 years or younger, accounting for 81% (n=17) of the studies. Only 19% (n=4) of the studies included participants who were older than 65 years.

The studies encompassed a wide range of cancer types and stages of cancer treatment (Table 1). Organ-specific cancers were the focus of 67% (n=14) of the studies, including breast cancer, head and neck cancer, lung cancer, and various others. The remaining 33% (n=7) of the studies did not specify the type of cancer, focusing instead on survivors of cancer or patients with cancer undergoing chemotherapy or radiotherapy. In total, 6 (29%) studies included participants who were in the posttreatment, while 3 (14%) studies involved participants undergoing treatment. Only 2 (10%) studies included participants before the start of the treatment, and 10 (48%) studies had unclear stages of treatment.

### Technology Used

As shown in Table 2, the papers included in this scoping review used various technologies to deliver exercise programs or monitor participants, highlighting the diverse approaches to telerehabilitation for patients with cancer. Most studies (n=13, 62%) used web-based systems, such as Retwise, e-CUIDATE, and SwallowIT, to facilitate patient and provider interactions. Mobile apps were used in 24% (n=5) of the studies, with apps like Physitrack (Physitrack PLC), Second Wind (Mediplus Solution), and the BENECA mobile health (mHealth) app (Mixed University Sport and Health Institute) being notable examples.

**Table 2:** Intervention characteristics.

Paper	Technology used to deliver exercise programs or monitoring	Type of exercise program	Exercise program description	Duration, frequency, time per session, and intensity of the program	Monitoring of performance	Outcomes measured
Schwartz et al (2015) [30]	Web-based system (Retwise website) for patient+pulse oximeter	In-person clinic-based rehabilitation+self-directed home-based tailored exercise program	Aerobic and resistance training.	12 weeks, 3-4 sessions per week, 20 minutes of aerobic exercise at an intensity of 60%-70% of aerobic capacity, and 3-5 resistance exercises with unclear time per session, neither intensity.	Self-monitoring using digital tools and web system	6MWT^a^, 1-repetition maximum of lower and upper body strength.
Galiano-Castillo et al (2016) [31]	Web-based system (e-CUIDATE website) for patient and provider+phone call	Home-based remote real-time guidance provided by CUIDATE research staff	(1) Warm‐up, (2) resistance and aerobic exercise training, and (3) cool‐down.	8 weeks, 3 sessions per week, 90 minutes per session. Intensity and volume of exercise according to guidelines of the American College of Sports Medicine for survivors of cancer.	Remote asynchronous and synchronous monitoring via web system, videoconferencing, or phone calls, on-demand by CUIDATE research staff	QoL^b^, Brief Pain Inventory, handgrip dynamometer, isometric abdominal test, back dynamometer, multiple sit‐to‐stand test, and the Piper Fatigue Scale.
Collins et al (2017) [32]	Web-based system (unspecified website) for patient and provider	Home-based remote real-time guidance provided by clinic staff	Rehabilitation of swallowing and communication function, nutritional management, and review of posttreatment symptoms.	8 months, unclear frequency, neither time per session, and these were requested on-demand. Unclear intensity.	Unclear	Service outcomes, costs, and consumer satisfaction.
Galiano-Castillo et al (2017) [33]	Web-based system (e-CUIDATE website) for patient and provider+phone calls	Web system–guided home-based tailored exercise program	(1) Warm‐up, (2) resistance and aerobic exercise training, and (3) cool‐down.	8 weeks, 3 sessions per week, 90 minutes per session. Intensity and volume of exercise according to guidelines of the American College of Sports Medicine for survivors of cancer.	Remote asynchronous and synchronous monitoring via web system, videoconferencing, or phone calls, on-demand by CUIDATE research staff	6MWT, Auditory Consonant Trigrams, and Trail Making Test.
Wall et al (2017) [34]	Web-based system (SwallowIT website) for patient and provider	Web system–guided home-based tailored exercise program	Swallowing exercises based on the “Pharyngocise” protocol.	6 weeks, daily, 45 minutes per session. Unclear intensity.	Remote asynchronous monitoring after exercise via web system, unclear frequency by the speech pathologist	Perceptions were evaluated via structured questionnaires and phone interviews. Patients’ perceptions toward using SwallowIT (4 questions), the functionality of the system (2 questions), the efficacy of the system (4 questions), and preferences for other service-delivery models (2 questions).
Frensham et al (2018) [35]	Web-based system (STRIDE website) for patient+pedometer	Self-directed home-based tailored exercise program	Individual target steps per day program.	Unclear.	Self-monitoring via web system, daily	Measures of physiology, physical ﬁtness, QoL, and 6MWT.
Gehring et al (2018) [36]	Web-based system (unspecified website) for patient and provider+HR^c^ monitor watch+phone calls	Self-directed home-based tailored exercise program	The intervention comprised 3 home-based aerobic training sessions per week for 6 months.	6 months, 3 sessions per week, unclear time per session. Intensity of 60%-85% of maxHR.	Remote asynchronous monitoring after exercise via the system weekly by the physiotherapist	Feasibility (accrual, attrition, adherence, and safety), satisfaction, patient-reported physical activity, VO_2_ peak^d^, and BMI.
Vallerand et al (2018) [37]	Phone call–based system for both patients and providers	Self-directed home-based regular progressing exercise program	Aerobic exercises.	12 weeks, unclear frequency, recommended 60-300 minutes per week time per session. Unclear intensity.	Remote synchronous monitoring or coaching via phone call weekly by research staff	Self-reported aerobic exercise behavior, QoL, fatigue, and program satisfaction. Feasibility metrics (recruitment, adherence, adverse events, retention, follow-up, and acceptability metrics).
Villaron et al (2018) [38]	Pedometer+SMS text messaging	Self-directed home-based standard exercise program	Walking program with a pedometer.	8 weeks, unclear frequency, time per session, neither intensity.	Remote asynchronous coaching, weekly by research staff	Level of physical activity (pedometer), fatigue (MFI-20^e^), and EORTC-QLQ-C30^f^.
Cheville et al (2019) [39]	Web-based system (unspecified website) for both patient and providers+pedometer+phone call	Self-directed home-based tailored exercise program	The physical therapists instructed patients in an incremental pedometer–based walking program and a resistive exercise program.	6 months, recommended at least 4 sessions per week, unclear time per session, neither intensity.	Remote synchronous monitoring after exercise via phone call, on demand by physiotherapist Remote asynchronous monitoring via web system, weekly by physiotherapist	Activity measure (computer adaptive test), pain interference and average intensity (Brief Pain Inventory), and QoL (EQ-5D-3L).
Ji et al (2019) [40]	Mobile app (efil breath) for patients+wearable pulse oximeter+web-based system for providers	Mobile app–guided home-based tailored or fixed exercise program	Walking distance exercise program mainly and resistance exercises guidance videos.	12 weeks, unclear frequency, time per session, neither intensity.	Remote asynchronous monitoring after exercise via web system, unclear frequency by lung cancer specialists and nurses	6MWT, dyspnea (mMRC^g^), QoL (EQ-5D), and service satisfaction.
Longacre et al (2019) [41]	Web-based system (unspecified website) for both patient and providers+pedometer+phone call	Self-directed home-based tailored exercise program	Pedometer-based walking program and a resistive exercise program.	6 months, recommended at least 4 sessions per week, unclear time per session, neither intensity.	Remote synchronous monitoring after exercise via phone call, on demand by physiotherapist Remote asynchronous monitoring via web system, weekly by physiotherapist	QoL (EQ-5D-3L) and intervention costs.
van Egmond et al (2020) [42]	Mobile app (Physitrack) for patients	Web system–guided home-based tailored exercise program	Muscle strength, coordination, range of joint motion, and stamina.	12 weeks, at least 2 sessions per week, unclear time per session. The intensity and frequency of the functional exercises were determined according to the guidelines of the American College of Sports Medicine.	Remote synchronous monitoring after exercise via phone call, SMS text messaging, or videoconference weekly by physiotherapist	Willingness, adherence, refusal rate, treatment duration, occurrence of adverse events, patient satisfaction. Musculoskeletal and cardiovascular functions and activities.
Kim et al (2020) [43]	Mobile app (Second Wind) for patients and providers+IoT^h^ track device (HR, steps, calorie expenditure, and exercise time)	Mobile app–guided home-based tailored exercise program	Warm-up, stretching, aerobic, and muscle-strengthening exercises for the upper and lower extremities.	12 weeks, unclear frequency, neither time per session. Intensity and target HR for the aerobic exercise were set from the results of the 6MWT.	Self-monitoring using digital tools and on-demand remote asynchronous monitoring by the study coordinator	6MWT, grip strength test, 30-second chair stand test, IPAQ-SF^i^, body composition, biochemical profiles, and QoL (C30).
MacDonald et al (2020) [44]	Mobile app (Physitrack) for patients and providers+Fitbit+phone calls	Mobile app–guided home-based tailored exercise program	Aerobic exercise for 150 minutes per week, 2-3 days of resistance training, and routine large muscle group ﬂexibility training.	8 weeks, 2-3 sessions per week, unclear time per session, neither intensity.	Self-monitoring via mHealth^j^ app and remote asynchronous monitoring via web system and feedback provided via phone call weekly by kinesiologist	Feasibility, acceptability. Physical symptoms, social functioning, distress, physical activity, work function, and physiological factors.
Piraux et al (2020) [45]	Web-based system (Virtuagym website) for patients and provider+phone calls	Digital tool–guided home-based tailored exercise program	Tele-prehabilitation, including aerobic, resistance, and inspiratory muscle training.	2-4 weeks, 3-5 sessions per week, 75 minutes per session. Intensity of 65%-74% of maximum HR for aerobic exercises.	Remote synchronous monitoring after exercise via phone call by physiotherapist	Feasibility (recruitment rate, retention rate, attendance to exercise sessions, exercise-related adverse events, and patient satisfaction), 6MWT, fatigue, QoL, anxiety, and depression.
Zhou et al (2021) [46]	Virtual reality–based system	By design, digital tool–guided home-based tailored program	(1) Fist clenching, (2) wrist twisting, (3) elbow bending, (4) lifting, (5) shoulder circling, (6) ear touching, (7) wall climbing, (8) backhanding, (9) head holding, (10) abduction.	1 session.	Unclear	General information questionnaire, usability surveys: System Usability Scale (SUS), SSQ^k^, and PQ^l^.
Finkelstein et al (2022) [47]	Web-based system (HAT system website) for patients	By design, web system–guided home-based tailored program	Individuality: specific exercises based on patients’ needs.	1 session.	Remote asynchronous monitoring after exercise via system by the health provider	Surveys: sociodemographic form, the Rapid Estimate of Adult Literacy in Medicine, SUS; semistructured qualitative exit interview.
Lozano-Lozano et al (2020) [48]	Mobile app (BENECA mHealth app) for patients	In-person clinic-based rehabilitation	Individualized AROM^m^ session.	8 weeks, 3 sessions per week, 75-95 minutes per session. Unclear intensity.	Self-monitoring via mHealth app	QoL (EORTC QLQ-C30 and EORTC QLQ-BR23^n^), Disabilities of the Arm, Shoulder, and Hand (DASH), a self-reported questionnaire that measures symptoms and physical function (disability) for any upper-limb region.
Park et al (2023) [49]	Virtual reality–based system (Kinnect motion capture via Xbox [UINCARE Home+rehabilitation system])	Digital tool–guided home-based tailored exercise program	Each exercise level was composed of warm-up (deep breathing+trunk twist), main workouts (different degrees of motion and variations of passive or active flexion, rotation, and abduction exercises with or without dumbbells were used), and cool-down (deep breathing) components. The exercise level was determined according to the results obtained over the first 4 weeks. Passive and active ROM^o^ of shoulder exercises were included.	12 weeks, daily, unclear time per session, neither intensity.	Remote asynchronous monitoring after exercise via a system by the physician	ROM of the affected shoulder, pain in the affected shoulder (Numerical Rating Scale), functional outcomes (Quick DASH score), and QoL (Functional Assessment of Cancer Therapy-Breast and EQ-5D-5L).
Filakova et al (2023) [50]	Web-based system (PolarFlow website) for patient+HR monitor sync to website+phone call	Self-directed home-based tailored exercise program	Modality of walking, Nordic walking, or cycling dependent on patient preference.	12 weeks, 3 sessions per week, 30-50 minutes per session. Intensity of 60%-85% HRmax and 11-13 on the Borg rating of RPE^p^.	Remote synchronous monitoring after exercise via phone call weekly by the physiotherapist Remote asynchronous monitoring via web system, unclear frequency by physiotherapist	Weight, body composition, cardiopulmonary exercise test.

^a^6MWT: 6-minute walking test.

^b^QoL: quality of life.

^c^HR: heart rate.

^d^VO_2_ peak: peak oxygen uptake.

^e^MFI-20: Multidimensional Fatigue Inventory.

^f^EORTC QLQ-C30: European Organization for Research and Treatment of Cancer Quality of Life Questionnaire Core 30.

^g^mMRC: modified Medical Research Council Dyspnea Scale.

^h^IoT: Internet of Things.

^i^IPAQ-SF: International Physical Activity Questionnaire-Short Form.

^j^mHealth: mobile health.

^k^SSQ: Simulator Sickness Questionnaire.

^l^PQ: Presence Questionnaire.

^m^AROM: active range of motion.

^n^EORTC QLQ-BR23: European Organization for Research and Treatment of Cancer Quality of Life Questionnaire and Breast Module.

^o^ROM: range of motion.

^p^RPE: rate of perceived exertion.

Phone call or SMS text messaging–based systems were used in 43% (n=9) of the studies, either as stand-alone methods or in conjunction with other technologies. For instance, Vallerand et al [37] and Villaron et al [38] used phone calls and SMS text messaging, respectively, to deliver and monitor exercise programs. Additionally, medical devices were integrated into 24% (n=5) of the studies, often paired with other technologies. Examples include pulse oximeters, pedometers, and heart rate monitor watches.

Immersive technologies, such as virtual reality (VR), were used in 10% (n=2) of the studies. These included systems like the Kinect motion capture via Xbox and other VR-based approaches.

The studies varied in the number of technologies used. Approximately 48% (n=10) of the studies used only 1 type of ICT to deliver their programs. In contrast, 9 (43%) studies used 2 types of ICT, combining methods like web-based systems with phone calls or medical devices. A smaller portion (n=2, 10%) used 3 types of ICT.

Several studies combined different technologies to enhance the delivery and monitoring of exercise programs. For example, Ji et al [40] used a combination of a mobile app (efil breath; LifeSemantics Corp), a wearable pulse oximeter, and a web-based system for providers. Similarly, MacDonald et al [44] integrated a mobile app (Physitrack), a Fitbit device, and phone calls to provide comprehensive patient support. Other studies focused on leveraging the strengths of specific technologies. For instance, van Egmond et al [42] used the mobile app Physitrack for patient engagement, while Finkelstein et al [47] used the Home Automated Telemanagement website to facilitate patient interactions.

### Exercise Program Details

Most physical rehabilitation programs (n=7, 33%) were self-directed, home-based tailored exercise programs, where patients followed individualized exercise plans independently. Web system–guided programs accounted for 24% (n=5) of the studies, using digital platforms to provide real-time or asynchronous guidance. Mobile app–guided programs comprised 14% (n=3) of the studies, leveraging mHealth apps to deliver and monitor exercise routines. Additionally, 14% (n=3) of the programs were directly guided by health providers, and digital tools guided 10% (n=2).

Most exercise programs (n=19, 90%) were home-based, enabling patients to perform their routines in a familiar environment. Only 1 (5%) study included clinic-based rehabilitation, and another (n=1, 5%) combined home and clinic-based exercises. The types of exercises predominantly included aerobic (n=19, 90%), resistance (n=13, 62%), and flexibility training (n=7, 33%). Only 2 (10%) studies focused explicitly on swallowing exercises, addressing particular needs of patients with oropharyngeal cancer.

The duration of the exercise programs varied, with 11 (52%) of the papers reporting interventions extending beyond 2 months and 7 (33%) lasting 2 months or less. The frequency of exercise sessions was less than daily in 48% (n=10) of the studies, while daily exercise was prescribed in 14% (n=3). However, the exercise frequency was unclear in 29% (n=6) of the studies. The time per session was varied, with 24% (n=5) of the studies specifying sessions of 1 hour or less and 10% (n=2) indicating sessions longer than 1 hour. The time per session was unclear in 57% (n=12) of the studies. The exercise intensity was explicitly defined in 38% (n=8) of the studies, while it remained unclear in 52% (n=11).

Monitoring methods were diverse, reflecting the integration of various technologies and approaches. Remote asynchronous monitoring was common, with many studies using web systems, phone calls, or mobile apps to track patient progress. For instance, Galiano-Castillo et al [31,33] used both synchronous and asynchronous monitoring via web systems and videoconferencing, while MacDonald et al [44] combined self-monitoring via a mHealth app with weekly feedback from a kinesiologist. Self-monitoring was also a key component in several programs. Schwartz et al [30] and Kim et al [43] implemented self-monitoring using digital tools, allowing patients to track their own progress and report it to health care providers as needed.

### Outcomes Measured

The outcomes measured in the studies included in this scoping review highlight the multifaceted approach to assessing the effectiveness and feasibility of physical telerehabilitation programs for patients with cancer. These outcomes can be broadly categorized into QoL, usability, feasibility, and functional outcomes, with some studies measuring additional specific outcomes.

QoL was a key outcome measured in 48% (n=10) of the studies. Instruments such as the EQ-5D-3L, Brief Pain Inventory, Piper Fatigue Scale, and various cancer-specific QoL questionnaires like the European Organization for Research and Treatment of Cancer Quality of Life Questionnaire Core 30 were commonly used. For instance, Galiano-Castillo et al [31] and Cheville et al [39] used these tools to evaluate participants’ overall well-being and health status, while van Egmond et al [42] assessed musculoskeletal and cardiovascular functions and activities alongside patient satisfaction.

Usability outcomes were assessed in 38% (n=9) of the studies, focusing on the practicality and user-friendliness of the telerehabilitation interventions. Studies like those by Wall et al [34] and Finkelstein et al [47] used structured questionnaires and surveys, including the System Usability Scale, to gather feedback on participants’ experiences and satisfaction with the technological platforms used.

Feasibility outcomes, measured in 14% (n=3) of the studies, included metrics such as recruitment rates, adherence, retention, and safety. The studies by Gehring et al [36] and MacDonald et al [44] focused on these aspects to determine the practicality and acceptability of the interventions.

Functional outcomes were the most frequently assessed, with 71% (n=15) of the studies measuring various aspects of physical performance. Commonly used measures included the 6-minute walk test, grip strength tests, and body composition assessments. Studies like those by Schwartz et al [30] and Kim et al [43] used these tests to evaluate improvements in physical fitness and functional capacity. Additionally, specific functional outcomes related to cancer treatment, such as the Disabilities of the Arm, Shoulder, and Hand questionnaire used by Lozano-Lozano et al [48], were also assessed.

Other outcomes measured in 33% (n=7) of the studies included service outcomes, costs, and consumer satisfaction, as seen in the study by Collins et al [32]. Additionally, some studies measured unique outcomes specific to the intervention or population, such as weight and body composition, as in the study by Filakova et al [50].

Most studies (n=11, 52%) measured 2 outcomes, integrating assessments of functional performance and QoL or usability. For example, Ji et al [40] evaluated the 6-minute walk test, dyspnea, QoL, and service satisfaction, providing a comprehensive overview of the intervention’s impact. A smaller portion of studies (n=5, 24%) measured 3 or more types of outcomes, offering a detailed evaluation across multiple dimensions.

## Discussion

### Principal Results and Comparison With Other Studies

This scoping review aimed to explore the existing telerehabilitation studies for patients with cancer. We included 21 papers that met our criteria. The major findings indicated that physical therapy delivered via telehealth for patients with cancer can improve functional capacity, cognitive functioning, and QoL [33,48]; reduce pain and hospital length of stay [39]; and improve fatigue, physical well-being, emotional well-being, and anxiety [45]. Additionally, improvements in absolute peak oxygen uptake and BMI [36,50]; handgrip strength of affected and nonaffected sides; abdominal, back, and lower body strength [31]; physical ﬁtness, systolic blood pressure, diastolic blood pressure, waist girth, mental health, social functioning, and general health [35]; and strength and endurance were observed [30]. Positive effects on feasibility [32,36,37,42,44-47], acceptability [30,34,44], and cost-effectiveness were also noted [41].

These findings align with previous studies demonstrating the feasibility of physiotherapy with telerehabilitation. For instance, a systematic review with meta-analysis by van Egmond et al [51] showed that telerehabilitation in surgical populations is feasible and can enhance QoL. Given that the effectiveness of telerehabilitation is at least equal to usual care for physical outcomes, it presents a viable alternative for physical therapy [51]. The improvement of QoL was a major outcome across most studies; similarly, a systematic review by Bártolo et al [52] found a trend toward improved QoL among patients with cancer who were exposed to telecare interventions.

This review included 10 RCTs, 7 feasibility studies, and 4 usability studies. Consequently, there is a need for more robust studies on cancer telerehabilitation, with greater uniformity in clinical trial reports. Developing clinical practice guidelines and integrating exercise and rehabilitation services into the cancer care delivery system are essential steps forward [53].

Research indicates that exercise is advantageous before, during, and after cancer treatment, applicable to all cancer types and various cancer-related impairments [53]. Engaging in moderate to vigorous exercise is particularly effective for enhancing physical function and alleviating cancer-related impairments. Supervised exercise programs have been shown to provide greater benefits than unsupervised ones, with serious adverse events being rare [53]. In our review, the exercises included aerobic routines, resistance training, swallowing exercises, and walking programs, all supervised via web-based systems, mobile apps, and telephone calls.

However, our review also reveals gaps in the current literature, particularly in the underreporting of exercise intensity and frequency, which are crucial for understanding the full impact of these programs. Future studies should provide more detailed descriptions of these parameters to enhance the reproducibility and comparability of findings. Moreover, while our review indicates overall positive outcomes, the variability in study designs and sample sizes suggests a need for more standardized methodologies to strengthen the evidence base.

A recent systematic review on the effectiveness of exercise-based telerehabilitation for patients with cancer demonstrated significant improvements in cardiorespiratory fitness (standardized mean difference=0.34; 95% CI 0.20-0.49) and physical activity (standardized mean difference=0.34; 95% CI 0.17-0.51) [54]. However, the review did not find significant changes in other outcomes, such as QoL, fatigue, or mental health. These findings underscore specific areas of measurable improvement while highlighting gaps in other critical domains of patient well-being. Complementarily, our scoping review uniquely contributes to this field by offering a more comprehensive examination of telerehabilitation interventions. Unlike the systematic review, we included quasi-experimental studies and assessed feasibility and usability outcomes, providing a broader understanding of the preliminary research landscape. This inclusive approach not only explores the outcomes evaluated by the interventions but also evaluates their practical implementation and user experience. By detailing the various components and methodologies of telerehabilitation programs, our review extends the current knowledge base, emphasizing the multifaceted benefits and challenges of implementing these interventions for patients with cancer. This holistic perspective is crucial for developing more effective and user-centered telerehabilitation strategies in oncology care.

We only found 2 papers using immersive technologies, such as VR, with 1 RCT reporting beneficial outcomes for patients. This finding aligns with recent evidence suggesting that VR is feasible for telerehabilitation in other chronic conditions, such as chronic obstructive pulmonary disease and orthopedic diseases [55,56]. Given the recent increase in research on immersive technologies, VR in telerehabilitation is a promising area for future exploration [57].

Another noteworthy aspect of our review is that only 5 papers referenced the use of wearable devices to provide patients with objective measures of progress during their rehabilitation. Although limited in our review, wearable devices offer significant potential for remote monitoring. A systematic review found that wearables significantly increased physical activity levels in patients with cardiovascular diseases [58]. This suggests that wearable or noncontact sensors [52] could be effectively integrated into telerehabilitation programs to enhance patient monitoring and outcomes.

Finally, using artificial intelligence (AI) in telerehabilitation is a technological trend worth observing. Our review did not find any papers referencing the use of AI. Still, the recent exponential growth in AI applications in health care suggests this trend could be explored in future studies. AI has the potential to significantly impact telerehabilitation by providing personalized and adaptive interventions based on patient data [59,60]. Exploring AI integration could open new avenues for improving the effectiveness and efficiency of telerehabilitation programs.

### Limitations

This scoping review has some limitations that should be acknowledged. First, the heterogeneity of the included studies presents a challenge in synthesizing the findings. The studies varied widely in terms of their design, participant characteristics, types of cancer, interventions, and outcomes measured. This variability makes it difficult to draw definitive conclusions about the overall effectiveness of telerehabilitation for patients with cancer. Despite this, the diversity of studies also highlights the flexibility and adaptability of telerehabilitation interventions, which is a strength in addressing the varied needs of patients with cancer. Second, the reliance on self-reported data for some outcomes may introduce reporting bias and affect the accuracy of the findings. While self-reported measures are valuable for assessing subjective outcomes like QoL, they are susceptible to inaccuracies. Objective measures such as wearable devices to monitor physical activity and physiological parameters can help validate self-reported data and provide a more comprehensive assessment. Third, many of the included studies had relatively small sample sizes, limiting the statistical power and generalizability of the results. Conducting larger, multicenter studies would increase sample sizes and enhance the representativeness of the findings, providing more robust statistical power to detect significant effects. Fourth, the technological variability across studies, with different platforms used for delivering and monitoring telerehabilitation, adds another layer of complexity and affects the comparability of the results. Standardizing the technological platforms used in interventions could reduce variability and improve comparability. Fifth, our review did not include a formal risk of bias evaluation, which could affect the reliability of our conclusions. While we included RCTs and quasi-experimental studies, which generally have higher quality, and ensured that all studies came from peer-reviewed journals, future studies should incorporate a formal risk of bias assessment to further enhance the rigor and reliability of the findings. Finally, we acknowledge that this is a rapidly evolving field, and more recent studies or those published before 2015 may have been missed. Moreover, while we conducted a thorough search, the exclusive use of PubMed as the database and the specific term “telerehabilitation” may have limited the identification of some relevant papers. The term “telerehabilitation” is relatively recent and might not be uniformly used across different regions and research contexts, potentially omitting some studies that use alternative terminology. Future reviews could benefit from including multiple databases and a broader range of search terms to capture the full scope of the literature. Despite these limitations, our review provides a comprehensive overview of the current state of research in telerehabilitation for patients with cancer, highlighting important trends and gaps that can inform future studies and clinical practice.

### Conclusions

This scoping review demonstrates that telerehabilitation exercises for patients with cancer are beneficial and feasible, with various approaches used in study design, technology, exercises, and outcomes. The evidence indicates that telerehabilitation can improve functional capacity, cognitive functioning, QoL, and other health metrics while being cost-effective and acceptable to patients. However, the review also highlights significant variability in study designs and a need for more detailed reporting on exercise intensity and frequency. Future research should focus on developing standardized methodologies, incorporating objective measures, and exploring emerging technologies such as VR and AI to optimize telerehabilitation interventions for patients with cancer. By addressing these areas, we can enhance clinical practice and improve outcomes for remote rehabilitation with patients.

## Appendix

Multimedia Appendix 1 PRISMA-ScR (Preferred Reporting Items for Systematic Reviews and Meta-Analyses Extension for Scoping Reviews) checklist.

## Abbreviations

AI: artificial intelligence
ICT: information and communication technology
mHealth: mobile health
PRISMA-ScR: Preferred Reporting Items for Systematic Reviews and Meta-Analyses Extension for Scoping Reviews
QoL: quality of life
RCT: randomized controlled trial
VR: virtual reality

## References

[1] Siegel RL, Miller KD, Jemal A (2020). Cancer statistics, 2020. CA Cancer J Clin.

[2] Mustian KM, Sprod LK, Palesh OG, Peppone LJ, Janelsins MC, Mohile SG, Carroll J (2009). Exercise for the management of side effects and quality of life among cancer survivors. Curr Sports Med Rep.

[3] Schmitz KH, Courneya KS, Matthews C, Demark-Wahnefried W, Galvão DA, Pinto BM, Irwin ML, Wolin KY, Segal RJ, Lucia A, Schneider CM, von Gruenigen VE, Schwartz AL (2010). American College of Sports Medicine roundtable on exercise guidelines for cancer survivors. Med Sci Sports Exerc.

[4] Cieza A, Causey K, Kamenov K, Hanson SW, Chatterji S, Vos T (2021). Global estimates of the need for rehabilitation based on the Global Burden of Disease study 2019: a systematic analysis for the Global Burden of Disease Study 2019. Lancet.

[5] Bourgeois A, Horrill TC, Mollison A, Lambert LK, Stajduhar KI (2023). Barriers to cancer treatment and care for people experiencing structural vulnerability: a secondary analysis of ethnographic data. Int J Equity Health.

[6] Jiang C, Yabroff KR, Deng L, Wang Q, Perimbeti S, Shapiro CL, Han X (2022). Self-reported transportation barriers to health care among US cancer survivors. JAMA Oncol.

[7] Ackerman MJ, Filart R, Burgess LP, Lee I, Poropatich RK (2010). Developing next-generation telehealth tools and technologies: patients, systems, and data perspectives. Telemed J E Health.

[8] Fong KNK, Kwan RYC, Gu D, Dupre ME (2020). Telerehabilitation (remote therapy). Encyclopedia of Gerontology and Population Aging.

[9] Suso-Martí L, La Touche R, Herranz-Gómez A, Angulo-Díaz-Parreño S, Paris-Alemany A, Cuenca-Martínez F (2021). Effectiveness of telerehabilitation in physical therapist practice: an umbrella and mapping review with meta-meta-analysis. Phys Ther.

[10] Tchero H, Tabue Teguo M, Lannuzel A, Rusch E (2018). Telerehabilitation for stroke survivors: systematic review and meta-analysis. J Med Internet Res.

[11] Subedi N, Rawstorn JC, Gao L, Koorts H, Maddison R (2020). Implementation of telerehabilitation interventions for the self-management of cardiovascular disease: systematic review. JMIR Mhealth Uhealth.

[12] Keikha L, Maserat E, Mohammadzadeh Z (2022). Telerehabilitation and monitoring physical activity in patient with breast cancer: systematic review. Iran J Nurs Midwifery Res.

[13] Solomon RM, Dhakal R, Halpin SJ, Hariharan R, O'Connor RJ, Allsop M, Sivan M (2022). Telerehabilitation for individuals with spinal cord injury in low-and middle-income countries: a systematic review of the literature. Spinal Cord.

[14] Cox NS, Dal Corso S, Hansen H, McDonald CF, Hill CJ, Zanaboni P, Alison JA, O'Halloran P, Macdonald H, Holland AE (2021). Telerehabilitation for chronic respiratory disease. Cochrane Database Syst Rev.

[15] Truijen S, Abdullahi A, Bijsterbosch D, van Zoest E, Conijn M, Wang Y, Struyf N, Saeys W (2022). Effect of home-based virtual reality training and telerehabilitation on balance in individuals with Parkinson disease, multiple sclerosis, and stroke: a systematic review and meta-analysis. Neurol Sci.

[16] Baffert S, Hadouiri N, Fabron C, Burgy F, Cassany A, Kemoun G (2023). Economic evaluation of telerehabilitation: systematic literature review of cost-utility studies. JMIR Rehabil Assist Technol.

[17] Baigi SFM, Mousavi AS, Kimiafar K, Sarbaz M (2022). Evaluating the cost effectiveness of tele-rehabilitation: a systematic review of randomized clinical trials. Front Health Inform.

[18] Shaffer KM, Turner KL, Siwik C, Gonzalez BD, Upasani R, Glazer JV, Ferguson RJ, Joshua C, Low CA (2023). Digital health and telehealth in cancer care: a scoping review of reviews. Lancet Digit Health.

[19] Lippi L, Turco A, Moalli S, Gallo M, Curci C, Maconi A, de Sire A, Invernizzi M (2023). Role of prehabilitation and rehabilitation on functional recovery and quality of life in thyroid cancer patients: a comprehensive review. Cancers (Basel).

[20] Yang W, Du Y, Chen M, Li S, Zhang F, Yu P, Xu X (2023). Effectiveness of home-based telerehabilitation interventions for dysphagia in patients with head and neck cancer: systematic review. J Med Internet Res.

[21] Garavand A, Aslani N, Behmanesh A, Khara R, Ehsanzadeh SJ, Khodaveisi T (2023). Features of teleoncology in lung cancer: a scoping review. Patient Educ Couns.

[22] Chang P, Zheng J (2022). Updates in cancer rehabilitation telehealth. Curr Phys Med Rehabil Rep.

[23] Gonzalo-Encabo P, Wilson RL, Kang D, Normann AJ, Dieli-Conwright CM (2022). Exercise oncology during and beyond the COVID-19 pandemic: are virtually supervised exercise interventions a sustainable alternative?. Crit Rev Oncol Hematol.

[24] Deo SV, Pramanik R, Chaturvedi M, Nath A, Ghosh J, Das Majumdar SK, Salins N, Kadayaprath G, Garg PK, Chaturvedi A, Mathur S, Mathur P (2022). Telemedicine and cancer care in India: promises, opportunities and caveats. Future Sci OA.

[25] Skiba MB, Wells SJ, Brick R, Tanner L, Rock K, Marchese V, Khalil N, Raches D, Thomas K, Krause KJ, Swartz MC (2024). A systematic review of telehealth-based pediatric cancer rehabilitation interventions on disability. Telemed J E Health.

[26] Giustiniani A, Danesin L, Pezzetta R, Masina F, Oliva G, Arcara G, Burgio F, Conte P (2023). Use of telemedicine to improve cognitive functions and psychological well-being in patients with breast cancer: a systematic review of the current literature. Cancers (Basel).

[27] Arksey H, O'Malley L (2005). Scoping studies: towards a methodological framework. Int J Soc Res Methodol.

[28] Tricco AC, Lillie E, Zarin W, O'Brien KK, Colquhoun H, Levac D, Moher D, Peters MDJ, Horsley T, Weeks L, Hempel S, Akl EA, Chang C, McGowan J, Stewart L, Hartling L, Aldcroft A, Wilson MG, Garritty C, Lewin S, Godfrey CM, Macdonald MT, Langlois EV, Soares-Weiser K, Moriarty J, Clifford T, Tunçalp Ö, Straus SE (2018). PRISMA Extension for Scoping Reviews (PRISMA-ScR): checklist and explanation. Ann Intern Med.

[29] Gonçalves LRP, Finkelstein J (2024). Telerehabilitation for patients with cancer: a scoping review. Open Science Framework.

[30] Schwartz AL, Biddle-Newberry M, de Heer HD (2015). Randomized trial of exercise and an online recovery tool to improve rehabilitation outcomes of cancer survivors. Phys Sportsmed.

[31] Galiano-Castillo N, Cantarero-Villanueva I, Fernández-Lao C, Ariza-García A, Díaz-Rodríguez L, Del-Moral-Ávila R, Arroyo-Morales M (2016). Telehealth system: a randomized controlled trial evaluating the impact of an internet-based exercise intervention on quality of life, pain, muscle strength, and fatigue in breast cancer survivors. Cancer.

[32] Collins A, Burns CL, Ward EC, Comans T, Blake C, Kenny L, Greenup P, Best D (2017). Home-based telehealth service for swallowing and nutrition management following head and neck cancer treatment. J Telemed Telecare.

[33] Galiano-Castillo N, Arroyo-Morales M, Lozano-Lozano M, Fernández-Lao C, Martín-Martín L, Del-Moral-Ávila R, Cantarero-Villanueva I (2017). Effect of an internet-based telehealth system on functional capacity and cognition in breast cancer survivors: a secondary analysis of a randomized controlled trial. Support Care Cancer.

[34] Wall LR, Ward EC, Cartmill B, Hill AJ, Porceddu SV (2017). Examining user perceptions of SwallowIT: a pilot study of a new telepractice application for delivering intensive swallowing therapy to head and neck cancer patients. J Telemed Telecare.

[35] Frensham LJ, Parfitt G, Dollman J (2018). Effect of a 12-week online walking intervention on health and quality of life in cancer survivors: a quasi-randomized controlled trial. Int J Environ Res Public Health.

[36] Gehring K, Kloek CJ, Aaronson NK, Janssen KW, Jones LW, Sitskoorn MM, Stuiver MM (2018). Feasibility of a home-based exercise intervention with remote guidance for patients with stable grade II and III gliomas: a pilot randomized controlled trial. Clin Rehabil.

[37] Vallerand JR, Rhodes RE, Walker GJ, Courneya KS (2018). Feasibility and preliminary efficacy of an exercise telephone counseling intervention for hematologic cancer survivors: a phase II randomized controlled trial. J Cancer Surviv.

[38] Villaron C, Cury F, Eisinger F, Cappiello MA, Marqueste T (2018). Telehealth applied to physical activity during cancer treatment: a feasibility, acceptability, and randomized pilot study. Support Care Cancer.

[39] Cheville AL, Moynihan T, Herrin J, Loprinzi C, Kroenke K (2019). Effect of collaborative telerehabilitation on functional impairment and pain among patients with advanced-stage cancer: a randomized clinical trial. JAMA Oncol.

[40] Ji W, Kwon H, Lee S, Kim S, Hong JS, Park YR, Kim HR, Lee JC, Jung EJ, Kim D, Choi C (2019). Mobile health management platform-based pulmonary rehabilitation for patients with non-small cell lung cancer: prospective clinical trial. JMIR Mhealth Uhealth.

[41] Longacre CF, Nyman JA, Visscher SL, Borah BJ, Cheville AL (2020). Cost-effectiveness of the Collaborative Care to Preserve Performance in Cancer (COPE) trial tele-rehabilitation interventions for patients with advanced cancers. Cancer Med.

[42] van Egmond MA, Engelbert RHH, Klinkenbijl JHG, van Berge Henegouwen MI, van der Schaaf M (2020). Physiotherapy with telerehabilitation in patients with complicated postoperative recovery after esophageal cancer surgery: feasibility study. J Med Internet Res.

[43] Kim Y, Seo J, An S, Sinn DH, Hwang JH (2020). Efficacy and safety of an mHealth app and wearable device in physical performance for patients with hepatocellular carcinoma: development and usability study. JMIR Mhealth Uhealth.

[44] MacDonald AM, Chafranskaia A, Lopez CJ, Maganti M, Bernstein LJ, Chang E, Langelier DM, Obadia M, Edwards B, Oh P, Bender JL, Alibhai SM, Jones JM (2020). CaRE @ Home: pilot study of an online multidimensional cancer rehabilitation and exercise program for cancer survivors. J Clin Med.

[45] Piraux E, Caty G, Reychler G, Forget P, Deswysen Y (2020). Feasibility and preliminary effectiveness of a tele-prehabilitation program in esophagogastric cancer patients. J Clin Med.

[46] Zhou Z, Li J, Wang H, Luan Z, Li Y, Peng X (2021). Upper limb rehabilitation system based on virtual reality for breast cancer patients: development and usability study. PLoS One.

[47] Finkelstein J, Huo X, Parvanova I, Galsky M (2022). Usability inspection of a mobile cancer telerehabilitation system. Stud Health Technol Inform.

[48] Lozano-Lozano M, Martín-Martín L, Galiano-Castillo N, Fernández-Lao C, Cantarero-Villanueva I, López-Barajas IB, Arroyo-Morales M (2020). Mobile health and supervised rehabilitation versus mobile health alone in breast cancer survivors: randomized controlled trial. Ann Phys Rehabil Med.

[49] Park H, Nam KE, Lim J, Yeo SM, Lee JI, Hwang JH (2023). Real-time interactive digital health care system for postoperative breast cancer patients: a randomized controlled trial. Telemed J E Health.

[50] Filakova K, Janikova A, Felsoci M, Dosbaba F, Su JJ, Pepera G, Batalik L (2023). Home-based cardio-oncology rehabilitation using a telerehabilitation platform in hematological cancer survivors: a feasibility study. BMC Sports Sci Med Rehabil.

[51] van Egmond MA, van der Schaaf M, Vredeveld T, Vollenbroek-Hutten MMR, van Berge Henegouwen MI, Klinkenbijl JHG, Engelbert RHH (2018). Effectiveness of physiotherapy with telerehabilitation in surgical patients: a systematic review and meta-analysis. Physiotherapy.

[52] Bártolo A, Pacheco E, Rodrigues F, Pereira A, Monteiro S, Santos IM (2019). Effectiveness of psycho-educational interventions with telecommunication technologies on emotional distress and quality of life of adult cancer patients: a systematic review. Disabil Rehabil.

[53] Stout NL, Baima J, Swisher AK, Winters-Stone KM, Welsh J (2017). A systematic review of exercise systematic reviews in the cancer literature (2005-2017). PM R.

[54] Batalik L, Chamradova K, Winnige P, Dosbaba F, Batalikova K, Vlazna D, Janikova A, Pepera G, Abu-Odah H, Su JJ (2024). Effect of exercise-based cancer rehabilitation via telehealth: a systematic review and meta-analysis. BMC Cancer.

[55] Gabriel AS, Tsai T, Xhakli T, Finkelstein J (2023). Mixed-methods assessment of a virtual reality-based system for pulmonary rehabilitation. Stud Health Technol Inform.

[56] Lal H, Mohanta S, Kumar J, Patralekh MK, Lall L, Katariya H, Arya RK (2023). Telemedicine-rehabilitation and virtual reality in orthopaedics and sports medicine. Indian J Orthop.

[57] Pawassar CM, Tiberius V (2021). Virtual reality in health care: bibliometric analysis. JMIR Serious Games.

[58] Alam S, Zhang M, Harris K, Fletcher LM, Reneker JC (2023). The impact of consumer wearable devices on physical activity and adherence to physical activity in patients with cardiovascular disease: a systematic review of systematic reviews and meta-analyses. Telemed J E Health.

[59] Smiley A, Tsai T, Havrylchuk I, Gabriel A, Zakashansky E, Xhakli T, Lyu J, Cui W, Parvanova I, Finkelstein J (2024). Machine learning approaches for exercise exertion level classification using data from wearable physiologic monitors. Stud Health Technol Inform.

[60] Amjad A, Kordel P, Fernandes G (2023). A review on innovation in healthcare sector (telehealth) through artificial intelligence. Sustainability.

